# Chloroformic and Methanolic Extracts of *Olea europaea* L. Leaves Present Anti-Inflammatory and Analgesic Activities

**DOI:** 10.5402/2011/564972

**Published:** 2011-05-26

**Authors:** R Chebbi Mahjoub, M. Khemiss, M. Dhidah, A. Dellaï, A. Bouraoui, F. Khemiss

**Affiliations:** ^1^Laboratory of Physiology, Faculty of Dentistry, University of Monastir, Monastir 5000, Tunisia; ^2^Laboratory of Pharmacology, Faculty of Farmacry, University of Monastir, Monastir 5000, Tunisia

## Abstract

*Olea europaea* L. is used in traditional medicine in the Mediterranean areas. Its natural products are used in the treatment of different disorders, like fighting fever and some infectious diseases such as malaria, the treatment of arrhythmia, and relief of intestinal spasms. The aim of the current study is to investigate the possible anti-inflammatory and anatinociceptive effects of methanol and chloroformic extracts prepared from leaves of *Olea europaea* L. The anti-inflammatory and antinociceptive effects of the different extracts of *Olea europaea* leaves were assessed after intraperitoneal administration into rats and mice, using the carrageenan-induced paw edema model in rats to test the anti-inflammatory effect and the acetic acid-induced writhing in mice to test the analgesic effect. The chloroformic and methanolic leaves extracts, studied at the doses of 50, 100, and 200 mg/kg (Body Weight: BW), exhibited significant dose-dependent anti-inflammatory and analgesic activities. Based on the results obtained, it can be concluded that *Olea europaea* leaves extracts have anti-inflammatory and antinociceptive effects.

## 1. Introduction

The medicinal uses of different parts of *Olea europaea* L. have been known for a long time in all the countries of the Mediterranean basin. Olive oil was recognized as an important component of a healthy diet. Several epidemiological studies have shown that the incidence of coronary heart disease and certain cancers is low in the Mediterranean basin where the diet is rich in olive products [1, 2]. Historically, olive leaves have been used as a folk remedy for combating fevers and other diseases such as malaria. Previous studies showed that some extracts of this product decreased blood pressure in animals and increased blood flow in coronary arteries [3], relieved arrhythmia, and prevented intestinal muscle spasms [4]. 

A phytochemical investigation reported that oleuropein was isolated from the leaves of *Olea europaea* [5]. This compound is known for possessing a wide range of pharmacologic and health promoting properties including anti-arrhythmic, spasmolytic, immuno-stimulant, cardioprotective, hypotensive, antihyperglycemic, antimicrobial, and anti-inflammatory effects [6–8]. *Olea europaea* is also rich in other bioactive phenolic compounds that are reported to have anti-inflammatory activities [9] However, there is no sufficient scientific studies reporting the anti-inflammatory and analgesic activities of *Olea europaea* leaves. 

As a contribution to the study of the pharmacological activities of the different components of olive leaves, we searched the possible anti-inflammatory and antinociceptive effects of the chloroformic and methanolic olive leaves extracts using an in vivo experimental models, that is, the carrageenan-induced hindpaw edema for the anti-inflammatory activity and the acetic acid-induced abdominal writhing test for the analgesic activity. 

## 2. Materials and Methods

### 2.1. Plant Material and Extraction Procedure

Leaves of *Olea europaea* L. were collected from the region of Sfax in the south of Tunisia. A voucher specimen (No. 0506) was deposited in the herbarium of the botanic laboratory (Faculty of Pharmacy, Monastir, Tunisia). 

 The olive leaves were dried at room temperature and then powdered. The powder was extracted successively with chloroform and methanol by maceration at room temperature for 24 hours, and then it was filtered. The solvent was removed from resulting solution under vacuum in a rotary evaporator.

### 2.2. Phytochemical Screening

The different extracts of olive leaves were screened for iridoids, flavonoids, tannins, saponosides, anthracene heterosides, cardiotone heterosides and alkaloids.

### 2.3. Chemical Study by Nuclear Magnetic Resonance (NMR)

The analysis of our extract was carried out on a spectrometer “Bruker Avance-300 (300 MHZ).” The dimethylsulfoxyde (DMSO-d6) and the deuterium Chloroform (CDCl3) were used as solvents. The chemical movements (*δ*) are expressed in part per million (ppm).

### 2.4. Chemical Study by Infra-Red (IR)

IR spectrums were recorded on a spectrophotometer “Philips Anlytical PU 9800” in liquid film on pastills of sodium chloride. The frequencies of the absorption bands are expressed in cm^−1^.

### 2.5. Animals

Wistar rats (140–180 g) and Swiss albino mice (18–25 g) were obtained from Pasteur Institute (Tunis, Tunisia). They were all kept in polypropylene cages in a room under controlled condition. All animals were fed according to a standard diet ad libitum and had free access to drinking water.

### 2.6. Determination of Anti-Inflammatory Activity of Olea Leaves Extract

The carrageenan-induced hind paw edema model according to the method of Winter [10] was used to test the anti-inflammatory activity. The chloroformic extract was dissolved in Ethanol : Tween 80 : distilled water (0.5 : 0.25 : 4.25), and the methanolic extract was dissolved in the distilled water. The wistar rats were divided into groups of six animals each. The control group received 2.5 mL/kg (BW) of vehicle (saline solution or Ethanol-Tween80-distilled water 0.5 : 0.25 : 4.25). The standard group received acetyl salicylic acid (ASA) at a dose of 300 mg/kg (BW) or dexamethasone at a dose of 1 mg/kg (BW). The test groups received, intraperitoneally, the chloroformic and methanolic extracts at doses of 50, 100, and 200 mg/kg (BW). After thirty minutes, each rat was injected with 0.05 mL of 1% carrageenan suspension as a phlogistic agent into the subplantar tissue of the left hind paw. The paw volume, up to the tibiotarsal articulation, was measured using a plethysmometer (model 7140, Ugo Basile, Italy). The measures were taken at 0, 1, 2, 3, 4, and 5 hours after the carrageenan injection. The degree of inflammation was determined by the difference between the left and the right paw volumes. The percent of the edema's inhibition was calculated in comparison to the control groups.

### 2.7. Determination of Antinociceptive Activity of Olea Leaves Extract

The writhing test, a chemical visceral pain model, was chosen in this study to evaluate the antinociceptive effect and was based on the procedure which was described by Koster et al. [11]. Nociception was induced by an intraperitoneal injection of acetic acid to the mice. The animals were divided into groups of six mice each. The first group, a control group, was treated with 10 mL/kg (BW) of vehicle (saline solution or Ethanol : Tween 80 : distilled water 0.5 : 0.25 : 4.25) intraperitoneally. The second group, the standard group, was treated with ASA (200 mg/kg, BW), as a reference drug. The last group was treated intraperitoneally with the olive leaves extracts at different doses (50, 100, and 200 mg/kg, BW). 30 minutes after the administration of these different substances, all the animals received 10 mL/kg of 1% acetic acid intra-peritoneally. The number of abdominal writhes as a pain indicator was counted for 30 min, five minutes after the acetic acid injection. The antinociceptive activity was expressed as a percentage of inhibition of abdominal writhes.

### 2.8. Statistical Analysis

Data were expressed as mean ± S.E.M. Results were statistically evaluated using a one-way analysis of variance (ANOVA), followed by Dunnett's test. Results were considered significant when *P* < .05 (*P < .05; **P < .01; ***P < .001).

## 3. Results

### 3.1. Extracts Yield

The results of yield extracts are shown in Table 1.

### 3.2. Chemical Composition of Olea Leaves Extract

The preliminary phytochemical qualitative screening of the chloroformic and methnolic extracts of the leaves of *Olea europaea* revealed the presence of iridoids, flavonoids, and tannins as shown in Table 2.

### 3.3. NMR Analysis

The spectrums determined by NMR of our extracts allow us to detect peaks corresponding to different protons in Table 3.

### 3.4. IR Analysis

The spectrums determined by IR appear in the form of bands relative to the different functions in Table 4.

### 3.5. Anti-Inflammatory Effects of Olea Leaves Extract

**Figure 1:**
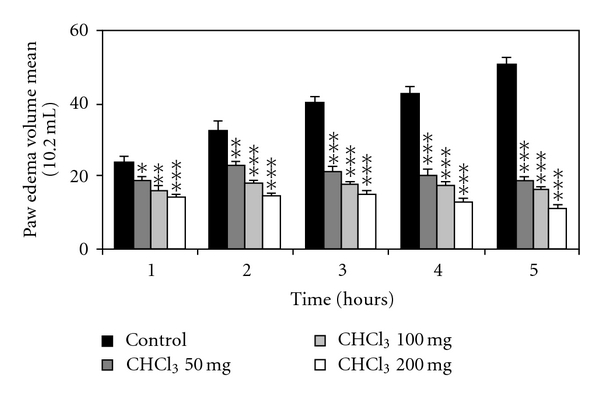
Effect of intraperitoneal administration of chloroformic (CHCl_3_) olive leaves extract on the carrageenan-induced rat hind paw edema. Values are mean ± SEM. *P < .05, **P < .01, and ***P < .001 versus control group, (*n* = 6).

**Figure 2:**
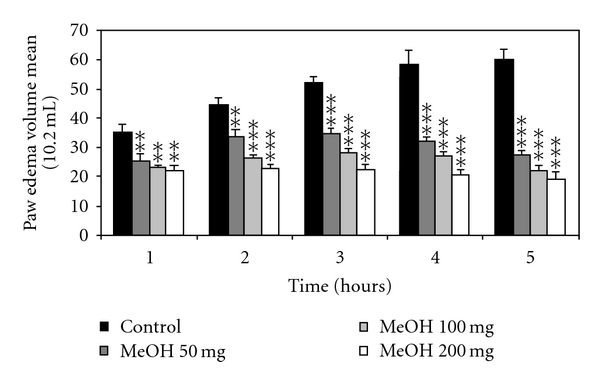
Effect of intraperitoneal administration of methanolic (MeOH) olive leaves extract on the carrageenan-induced rat hind paw edema. Values are mean ± SEM. *P < .05, **P < .01, and ***P < .001 versus control group, (*n* = 6).

**Figure 3:**
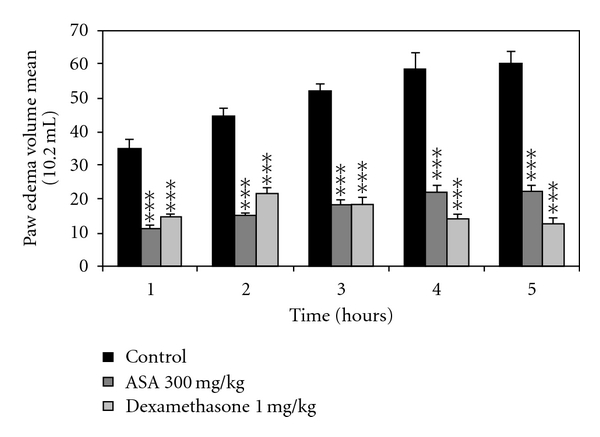
Effect of intraperitoneal administration of drug references (ASA and Dexamethasone) on the carrageenan-induced rat hind paw edema. Values are mean ± SEM. **P < .01, ***P < .001 versus control group, (*n* = 6).

The results reported in Figures 1, 2, and 3 showed the effect of the chloroformic and methanolic extracts of olive leaves (50, 100, and 200 mg/kg) administered intraperitoneally. These results revealed significant dose-dependent inhibitory effects of the carrageenan-induced paw edema at 1, 2, 3, 4, and 5 hours after injection. The effect appeared 1 hour after the injection and persisted all along the duration of the experiment (5 hours). The peak anti-inflammatory effect of the leaves extracts recorded with the dose of 200 mg/kg (BW) at the fifth hour was  77.8 ± 2% for the chloroformic extract and 68.1 ± 3.8% for the methanolic extract compared to the standard drugs (ASA and Dexamethasone) which decreased the paw edema by 62.9 ± 3.1% and 78.9 ± 3.3%, respectively, at the fifth hour.

### 3.6. Antinociceptive Effects of Olea Leaves Extract

**Figure 4:**
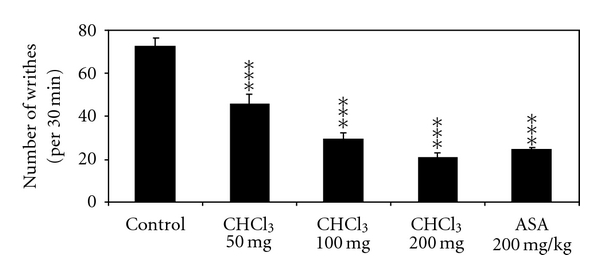
Effect of intraperitoneal administration of chloroformic (CHCl_3_) olive leaves extract on acetic acid-induced writhing in mice. Values are mean ± SEM. **P < .01, ***P < .001 versus control group, (*n* = 6).

**Figure 5:**
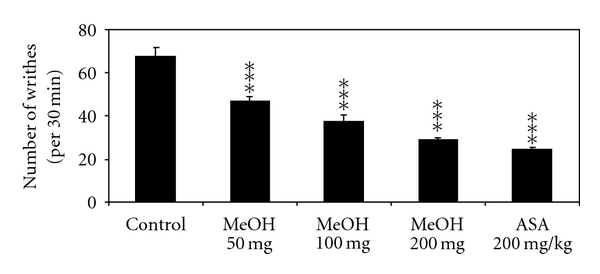
Effect of intraperitoneal administration of methanolic (MeOH) olive leaves extract on acetic acid-induced writhing in mice. Values are mean ± SEM. **P < .01, ***P < .001 versus control group, (*n* = 6).

The effects of the used extracts on writhing response in mice are reported in Figures 4 and 5. The intraperitoneal administration of the chloroformic and methanolic extracts at doses of 50, 100, and 200 mg/kg (BW) caused a significant reduction in the number of the writhing episodes induced by acetic acid compared to the control. The percentage of inhibition of the constrictions was calculated as 37.7 ± 6.8% (chloroformic extract 50 mg/kg), 60 ± 4.5% (chloroformic extract 100 mg/kg), 71.5 ± 3.5% (chloroformic extract 200 mg/kg), 31.4 ± 4% (methanolic extract 50 mg/kg), 44.9 ± 4.5% (methanolic extract 100 mg/kg), 57.3 ± 1.3% (methanolic extract 200 mg/kg), and 64.2 ± 2.1% (ASA). The 200 mg/kg dose of chloroformic extract was statistically highest than that of the reference drug ASA (*P* < .05).

## 4. Discussion

Both the chloroformic and methanolic extracts of olive leaves showed a significant anti-inflammatory effect in the acute phase of the inflammation process when compared with the standard anti-inflammatory drugs. The effect was observed from the first hour. The results showed that the anti-inflammatory activity of the chloroformic extract was more pronounced than the methanolic one. 

The in vivo acute inflammation model (Carrageenan-induced paw edema) has been frequently used to assess the anti-inflammatory effect of natural products [10, 12]. Several hypotheses have been advanced to explain the carrageenan mechanism action. In fact, various mediators are released by carrageenan in the rat paw. Thus, while the initial phase is mainly mediated by the release of histamine and serotonin, the second phase is due to the release of prostaglandins, protease, and lysosome [13–15]. Our results indicate that the olive leaves extracts significantly inhibit the development of carrageenan-induced rat paw edema. Similar results were noted in the presence of aqueous extract of citrillus colocynthis [16]. In addition, the mechanism of action may involve inhibition of production of proinflammatory cytokine (IL-6; IL-1*β*) and of the expression of COX-2 and at stimulation of the anti-inflammatory cytokine (IL-4) [17]. 

Interestingly, the anti-inflammatory effect of both tested extracts appears to be similar to that of dexamethasone, a steroidal anti-inflammatory drug, since in both cases the effect becomes more marked with time. In our experiment, we were limited to five hours after the injection of the phlogistic agent. It would be interesting to extend the time of our experimentation (24 h) to observe the effects of the extracts. 

For the antinociceptive activity, different tests were widely employed in mice such as p-benzoquinone and acetic-acid-induced writing tests and hot plate test [18, 19]. 

The olive leaves extract seems to exhibit a notable antinociceptive effects in the acetic acid-induced writhing test in the mouse. It is reported that acetic acid causes an increase in the peritoneal fluid levels of prostaglandins (PGE_2 _ and PGF_2*α*_), involving in part peritoneal receptors [20]. Prostaglandins may be involved in the antinociceptive action of the olive leaves extracts. 

The presence of iridoids and flavonoids in these organic extracts could be responsible for the anti-inflammatory and antinociceptive effects recorded in this study as it was reported earlier [16, 18, 21]. However, other results [22] suggest that the anti-inflammatory mechanism of ethanol extract of *Mahonia oiwakensis* may be related to the decrease of neutrophil infiltration, nitric oxide synthetase (iNOS), COX-2, protein expression, NO release, and TNF*α* level serum. 

Further studies are needed to isolate the different fractions in our global extract and to isolate and purify the active ingredients in the different fractions responsible for the anti-inflammatory and antinociceptive effects of *Olea europaea*.

## 5. Conclusion

Our current study is a first attempt to evaluate and validate the anti-inflammatory and analgesic activities of *Olea europaea* leaves confirming the traditional use of this plant in treating ailments associated with inflammation and pain. We suggest that the iridoids and flavonoids contents in olive leaves are major contributors to the anti-inflammatory and antinociceptive effects of olive leaves. A chemical study by NMR and IR of our extracts showed the presence of different compounds like hydrocarbon phenyl and ketone amine. These natural products are considered as active analgesic and anti-inflammatory ingredients [23, 24]. 

However, further detailed studies are required to determine the active ingredients responsible for these effects and to determine the mechanism of action of these compounds in the anti-inflammatory and analgesic processes.

## Figures and Tables

**Table 1:** Yield of different extracts.

	Extracts		
Results		Chloroformic extract	Methanolic extract
Mass (g)		31	60
Yield (%)		10,3	20

**Table 2:** Phytochemical screening of olive leaves extracts.

	Flavonoids	Iridoids	Tannins	Saponosids	Anthracenic heterosides	Cardiotonic heterosides	Alkaloïds
ChE	++	++	+	−	−	−	−
ME	+++	+++	++	−	−	−	−

(+): detectable and (−): not detectable. Abbreviations: Ch E: chloroformic extract; ME: methanolic extract.

**Table 3:** Different functions of *Olea europaea* determined by NMR analysis.

	Methanolic extract	Chloroformic extract
Type of proton	*δ* in ppm	*δ* in ppm
Radical phenyl	6.2 and 7.6	6 and 7.5
Alcohol	3.6	3.5
Amine	4.1	4.2
Hydrocarbon	2.4	1.3 and 1.5

**Table 4:** Different functions of *Olea europaea* determined by IR analysis.

	Methanolic extract	Chloroformic extract
Functions	Frequencies of bands in cm^−1^	Frequencies of bands in cm^−1^
-C=C-	1600	1600
-C=O-	1750	1700
Amine	3100–3250	2900–3250
Alcohol	3100–3250	2900–3250
